# Therapy of Sporadic and NF2-Related Vestibular Schwannoma

**DOI:** 10.3390/cancers12040835

**Published:** 2020-03-31

**Authors:** Longping Yao, Mohammed Alahmari, Yasin Temel, Koos Hovinga

**Affiliations:** 1 Department of Neurosurgery, Maastricht University Medical Center, 6202 AZ Maastricht, The Netherlands; loupe_yao@163.com (L.Y.); m.alahmari@maastrichtuniversity.nl (M.A.); y.temel@maastrichtuniversity.nl (Y.T.); 2 Department of Radiology, King Fahad Hospital of Imam Abdulrahman Bin Faisal University, P.O. Box 40046, 31952 AL-Khobar, Saudi Arabia

**Keywords:** vestibular schwannoma, NF2, management, surgery, radiotherapy, function preservation and radiation-induced effects

## Abstract

Vestibular schwannoma (VS) is a benign primary brain tumor that occurs sporadic or as part of a genetic syndrome. The most common cause is the mutation of the NF2 tumor suppressor gene that is involved in the production of the protein merlin. Merlin plays a role in cell growth and cell adhesion. In patients with NF2, the VSs arise bilaterally and coincide with other brain tumors. In sporadic VS, the tumor is typically unilateral and does not coincide in combination with other tumors. MRI is the standard imaging technique and can be used to assess the size and aspect of the tumor as well as the progression of disease. The preferred management of large VS in both VS types is surgery with or without adjuvant radiation. The management for the medium- or small-sized VS includes wait and scan, radiotherapy and/or surgery. This choice depends on the preference of the patient and institutional protocols. The outcomes of surgical and radiotherapy treatments are improving due to progress in surgical equipment/approaches, advances in radiation delivery techniques and dose optimizations protocols. The main purpose of the management of VS is preserving function as long as possible in combination with tumor control.

## 1. Introduction

Vestibular schwannoma (VS) is a benign tumor arising from Schwann cells of the vestibular part of the 8th cranial nerve (CN). VS is typically located in the cerebellopontine angle (CPA) and in approximately 10% of the cases it is located exclusively in the internal auditory canal (IAC). VS accounts for about 8%-10% of all intracranial tumors and for almost 75% tumors in the CPA [1]. The tumor can occur as a sporadic (isolated) lesion or as a part of a genetic syndrome. The majority of VS are sporadic (95%). In 5% of the VS cases the cause is neurofibromatosis type 2 (NF2) and the tumor occurs bilaterally [1,2]. The symptoms of VS largely depend on the compression of adjacent structures. The most common symptoms include progressive hearing loss, tinnitus, vertigo, ataxia and in the minority of the cases facial nerve (FN) dysfunction [2]. 

VS in NF2 patients is diagnosed much earlier than in sporadic cases due to autosomal dominant inheritance, which leads to screening of known patients and the presence of other symptoms causing brain tumors such as meningiomas, astrocytomas and ependymomas. The age of onset for genetic cases is at childhood and in sporadic VS around middle age [3,4]. Magnetic resonance imaging (MRI) is the standard imaging technique for diagnosing VS and can be used to assess the exact location, size, aspect of the tumor and progression of disease. The options of VS management mainly depend on the size and symptoms. There is a generally accepted classification for the size of tumor: small (less than 1.5 cm), medium (1.5–2.5 cm) or large (more than 2.5 cm). The preferred management of large VS in both VS types is surgery with or without adjuvant radiation. The management for the medium- or small-sized VS includes wait and scan, radiotherapy and/or surgery. This review will provide an update on the pathophysiology, imaging characteristics and discuss the latest insights in patient management.

## 2. Pathophysiology of VS

The molecular alterations of VS include NF2 gene mutation/loss/mitotic recombination [5,6,7], abnormal gene expression [8], involvement of immunological factors [9,10], deoxyribonucleic acid (DNA) methylation [11,12] and change in growth factors [13,14,15].

### 2.1. The Normal Structure and Function of the NF2 Gene

The NF2 gene encodes for a tumor suppressor protein, which is located on chromosome 22q12.2 [16,17]. The product of the NF2 gene, which is a 595-amino acid protein called the merlin protein, acts as a mediator in signal transmission [16,18,19]. It contains a relatively conserved N-terminal domain resembling the domain of ezrin-radixin-moesin protein family except for the C-terminal domain [20,21]. The domain is usually on the amino terminus, which allows it to act as a mediator to regulate cell motility, cell–cell attachment and cell membrane receptor availability [22]. Effectors to which merlin binds include PAK (p21-activated kinase), CD44, mTOR, Rac, Ras, Bcl-2 and interleukins. These effectors are involved in modulating cytoskeletal proteins or signal transduction of Rho family GTPases [20,23,24,25]. The phosphorylation of merlin is the main way to inhibit its activity on tumors [16].

### 2.2. Pathophysiology of Sporadic Form

Loss of merlin function is linked to the pathogenesis of sporadic VS [26,27,28]. Most of the mutations are point mutations and small deletions [22]. About 60% of the sporadic unilateral schwannomas have mutations in the NF2 gene [26,29]. To date, more than 200 mutations of NF2 have been identified. These include single-base substitutions, missense, insertions and deletions [30,31].

The “two-hit” hypothesis, also known as the Knudson hypothesis, implies that two mutations are needed to cause a phenotypic change. The term ‘‘hit” means a DNA mutation in a cell [32]. The “two-hit” inactivation of NF2 leads to the loss of merlin [33]. This theory also involves the loss of chromosome 22 and biallelic genetic alterations of the NF2 gene are seen in the majority of the sporadic VS cases [26]. Furthermore, the distinct merlin mutation has been shown in the most ‘one-hit’ schwannomas. Weak or non-bands were found in ‘two-hit’ tumors, showing that the inactivation of both alleles is necessary for the total loss of merlin expression. At least one somatic mutation occurred in more than 85% of the sporadic VS cases that affected the function of the NF2 gene [33].

Another possible mechanism for the inactivation of NF2 in sporadic VS is epigenetic modification. Cytosine phosphate guanine (CpG) islands are the stretches of the regions of the genome. CpG dinucleotides show higher density than other parts of the genome and CpG are normally unmethylated [34]. Methylation is a process to inhibit gene transcription at the transcriptional start site and 5′-UTR regions [11]. Methylation has been widely reported to be associated with VS. Gene promoter hypermethylation can silence transcription and terminate the expression of tumor suppresser genes [35]. Promoter methylation of NF2 is a key mechanism in Merlin expression and tumor development [14]. G/C are rich in the 5′and 3′ flanking regions of the human NF2 gene and sensitive to epigenetic factors [36]. Site-specific methylation of the promoter elements is the mechanism for inactivation of the NF2 gene in VS [37]. The methylation status of these specific sites is consistent with the expression of NF2 mRNA [37]. In VS, the methylation of the promoter-associated CpG island may be a secondary event of the inactivation of the gene in ‘one-hit’ tumors [17]. However, some researchers pointed out that the NF2 methylation is not a mechanism of merlin loss in schwannomas [38,39].

### 2.3. Pathophysiology of NF2 VS

NF2 is an autosomal dominantly inherited disorder. Mutations in the NF2 gene are responsible for NF2 VSs [40]. The patients can inherit the mutation from the affected allele of a parent or acquire a new mutation in the postzygotic stage of embryogenesis. Truncating mutations are associated with a severe clinical phenotype. Missense mutations and large deletions cause mild phenotypes [41,42,43,44].

The mutations are present in about 90% of the familial cases, which indicates that it can be used as a diagnostic tool when it is present in tissue and blood [42]. It has a major impact on individuals’ lives [43]. Some of the milder patients with NF2 VS have a mosaic mutation meaning that only some cells carry the mutation [45,46]. The risk of transmission to the next degeneration is therefore less than 50%. However, the offspring may show more severe symptoms than their parents since they will carry the mutation in all cells. Actually, somatic mosaicism for the NF2 gene is a frequently cause in the cases of new mutations (25%–30% of all NF2 cases) [47]. In some cases separate cell lines occur due to somatic mosaicism, one with the mutation and one without [48].

## 3. Imaging of VS

**Figure 1:**
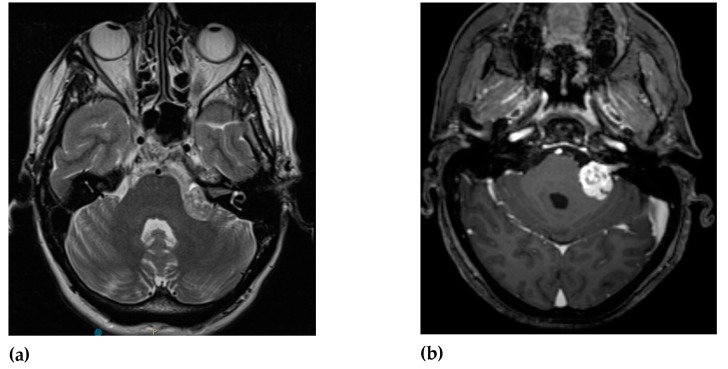
MRI imaging of sporadic vestibular schwannoma (VS) in cerebellopontine angle: (**a**) T2-weighted MRI reveals sporadic VS as hypointense signals and (**b**) T1-weighted image with contrast demonstrates hyperintensity of sporadic VS (courtesy of Y. Temel).

**Figure 2:**
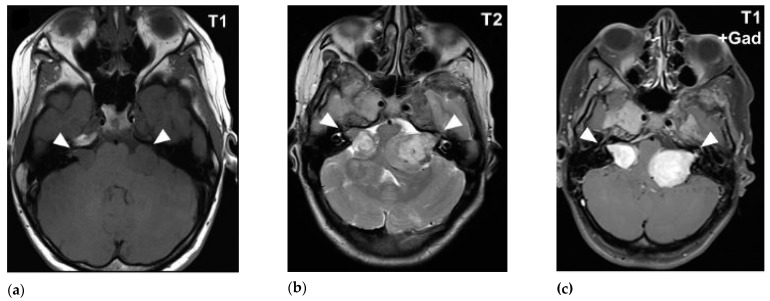
MRI imaging of bilateral NF2 VS in cerebellopontine angle: (**a**) T1-weighted MRI reveals bilateral NF2 VS as hypo-intense signals; (**b**) T2-weighted MRI and (c) T1-weighted MRI with contrast demonstrates hyperintensity of bilateral NF2 VS [53].

MRI is the standard imaging technique to assess the size, aspect, location and the evolution of VS. The ‘gold standard’ for diagnosing VS is the gadolinium-enhanced T1-weighted MRI sequence. Volume analysis using MRI imaging is preferred to evaluate the change in tumor size. A change of 20% in volume is considered clinically significant. Sometimes this can result in false-positive findings mainly with small lesion-like abnormalities [49,50]. T2-weighted MRI can help to show peritumoral oedema and the presence of cysts [51,52]. There are no major differences in MRI characteristics with routine imaging between NF2 VS and sporadic VS (Figure 1 and Figure 2). 

VS are hypo- to isointense or isointense when compared to brain parenchyma on T1-weighted images [54,55,56]. Tumoral cysts are hypointense in comparison to the brain parenchyma on T1-weighted images [54,57]. The T1-weighted images can visualize intratumoral hemorrhage as an isointense or hyperintense area [58,59]. On T2-weighted images VS shows hyperintense signals [55,60]. The cysts of VS are hyperintense and the tumor can appear heterogeneous when there is cystic degeneration [58,61]. Hemorrhage can be seen as a hypointense signal on T2-weighted images [58]. Intratumoral hemorrhage can occur in VS due to its high vascularity [1,62]. Since T2-weighted imaging has high resolution and lower costs it can be used as the follow-up modality [63].

## 4. Management of Sporadic VS and NF2 VS

The management VS depends on different factors such as size and growth of the tumor, symptoms associated with the tumor, patient preference and physician or institutional preferences. The main purpose of the management is preserving function as long as possible in combination with tumor control. Generally, the small-and medium sized VS can be managed with a wait and scan policy. When there is substantial growth radiotherapy can be considered. For large VS (of both VS types), with compression on the brainstem and demonstrable growth, the preferred treatment strategy is surgical resection with or without adjuvant radiation.

### 4.1. Sporadic VS

Function preservation of surrounding structures and mainly the 7th nerve is an important part of VS surgery. The surgical outcome in sporadic VS is associated with the following factors: tumor size, adhesion to the brainstem and CNs, the use of CN monitoring technology and surgeon’s experience [64,65,66,67,68,69]. Surgical treatments include subtotal resection (STR), near-total resection (NTR) and total resection [70]. Total resection provides a higher rate of long-term tumor control. However, it has a greater risk of permanent CN palsy [71,72]. Currently, the emphasis of VS surgical treatment has shifted from total tumor removal to function preservation. Thus, planned STR and NTR are applied more frequently and provide favorable preservation and recovery of FN function. If residual tumor grows during the follow-up radiotherapy is a good option [70,72]. In few cases, the tumor will continue to grow and requires a secondary treatment [73,74,75]. Chen et al. reported that the recurrence rate was 18% over an average follow-up of 3.8 years after STR [76]. Nakatomi et al. reported a 5% recurrence rate over an average follow-up of 5 years after NTR [74]. Currently, there is no tangible proof to state that one surgical approach is better than another approach. Therefore, it is important to develop an individualized treatment strategy for each patient based on evidence and institutional experience.

The routes to approach sporadic VS include transotic, translabrynthine (TL), middle cranial fossa (MCF) or retrosigmoid approach (RS). Transotic and TL will breach the inner ear apparatus leading to the loss of hearing function, which must be taken into consideration when selecting these approaches [77,78]. TL craniotomy allows access to the CPA directly and exposes the FN from brainstem to stylomastoid foramen through mastoidectomy and drilling of semicircular canals and vestibule [3,79]. It is applied to VS patients whose hearing function is poor or preservation of hearing is unlikely [80,81]. TL is the most direct route to access the CPA and expose IAC entirely [82,83]. These factors allow for the resection of larger size VS and gives a reliable potential plane between tumor and FN. However, when the size is greater than 3 cm in CPA, the surgical field is limited because of the restriction of external auditory canal and sigmoid sinus [8]. Changing the resection range of temporal bone can prevent cerebrospinal fluid leakage and preserve the FN function [81]. A functional amount of hearing preservation following the TL approach could be possible if preservation of the auditory division of 8th nerve is feasible [81]. Surgery is also frequently applied in NF2 associated ependymomas and meningiomas if they become symptomatic [82,83,84].

### 4.2. NF2 VS

The management of NF2 VS patients requires a multidisciplinary team. The resection of NF2 VS can be more difficult than sporadic ones because the tumor tends to be more adherent to adjacent structures [85]. The decision on the timing of surgical intervention is not an easy one. Some surgeons advocate that early surgical intervention is helpful to preserve hearing function and FN function [86,87]. In clinical practice, most NF2-related patients undergo surgical intervention when their tumors reach a size more than 2–3 cm [88]. One report showed that the diameters of tumors greater than 2.5 cm would increase the risk of FN injury with 17% [83]. However, the risk of bilateral hearing loss and FN damage is not negligible. It has been shown that the preservation rate of FN function was less than 50% and hearing preservation was successful in 3 of 11 cases in whose tumor was larger than 3 cm [89,90]. So, some neurosurgeons only recommend surgical intervention of these lesions when the tumor is large enough and has induced hearing loss, symptomatic brainstem compression or other serious symptoms [85,86]. Auditory brain stem implants and cochlear implantation may improve the outcome of hearing function [29,91,92].

The common surgical approach of NF2 VS is TL or RS [65,93]. TL is also applied in patients with large tumors even if functional hearing is present [87]. Middle fossa approach and RS may offer the possibility to maintain hearing function in NF2 patients [94]. MFA is applied for small- or mid-sized tumors. Table 1 summarized the comparison between sporadic VS and NF2 VS

## 5. Radiotherapy

The role of radiotherapy in VS and NF2 VS management is to stop the growth of the tumor and if possible preserving or gaining better functional outcomes. Radiotherapy has lower morbidity when compared to surgery [95,96]. Radiotherapy differs based on the physical properties of the radiation (photon-based or charged particles). Photon-based radiotherapy can be either administered by x-rays delivered by systems such as the linear accelerators (LINAC) and image-guided robotic system (CyberKnife) or in the form of gamma rays by using the Gamma knife (GK) system [97]. The most common examples for the charged or energetic particle therapies are proton and carbon-ion therapies [98]. In addition, the dose delivery technique can be in either single fraction stereotactic radiosurgery (SRS) or fractionated stereotactic radiotherapy (FSRT). Historically, the first and most widely used modality to treat VS is the Leksell Gamma Knife radiosurgery [99,100].

### 5.1. NF2 VS and Radiotherapy

Although it is widely accepted that radiotherapy is less effective in controlling NF2 VS than sporadic VS, the patient should be aware of radiotherapy as a management option; regardless of the tumor size [41,101,102]. Patients who refuse or are considered high-risk for surgery are considered good candidates for radiotherapy [2]. Outcomes are varying in the literature. Mathieu et al. have treated 62 of NF2 VS patients using GK radiosurgery with a dose range of 14–27.5 Gy. The mean tumor volume was 5.7 cm^3^ and serviceable hearing was present in 35% of the patients before treatment. The control and the hearing preservation rates were found to be 85%, 81% and 81% at 5, 10 and 15 years, and 73%, 59% and 48% at 1, 2 and 5-years respectively. The tumor volume was significantly predictive of local control [103]. Another group has shared their experience in treating 25 patients of NF2 VS (mean tumor diameter= 2.5 cm) with a linear accelerator [104]. Patients were irradiated in either a single fraction (mean dose= 10–12.5 Gy) or in five fractions (total mean dose= 20–25 Gy). Twenty patients were followed-up for more than one year and achieved 100% tumor control. Within 36 months, 12 out of 15 patients with serviceable hearing before the treatment lost less than 45 dB. Interestingly, no patient showed treatment-related toxicity in both facial and trigeminal nerves. Phi et al. reported the analysis of 36 NF2 VS patients who had undergone GK (median FU = 48.5 months) [105]. A mean marginal dose of 12.1 Gy was delivered to a mean tumor volume of 3.2 cm^3^. Five patients developed tumor recurrence and calculated control rates were 81%, 74% and 66% in the first, second, and fifth year, respectively. The hearing was preserved respectively in 50%, 45% and 33% (At the first, second and fifth year FU) out of 16 patients presented with a serviceable hearing before the treatment. In a recent systematic review comparing the outcomes of SRS and surgery in patients with NF2 VS, 485 patients received a single fraction of SRS were included in the pooled analysis. The mean control rate, hearing and FN preservation at 5-years were 75.1%, 40.1% and 92.3%, respectively [106]. Taken together, treating NF2 VS primarily with radiotherapy suggests that this treatment works best for tumor control and FN preservation and has a minor effect on hearing preservation.

### 5.2. Sporadic VS and Radiotherapy 

#### 5.2.1. Primary Radiotherapy in Small to Medium VS

Due to the benign and slow growing nature of VS, the choice of having an intervention in small to medium sized of VS is determined by the preference of the patient or the treatment team. In a systematic review aimed to compare outcomes of surgery and SRS with no earlier intervention, the pooled analysis demonstrated that SRS is superior to surgery in VS with a diameter less than 30 mm [107]. Both approaches were comparable in terms of tumor control. However, SRS was associated with better FN function, hearing preservation and quality of life with no observed mortalities when compared to surgery. A recent cohort study by Golfinos et al. compared the outcomes of SRS and surgery in VS patients (tumor diameter ≤ 28 mm). No differences were found in terms of functional outcomes, tumor control and mortalities [108]. Long-term follow-up comparisons between SRS and surgery are still lacking. Nevertheless, for smaller tumors SRS is the preferred treatment strategy in most skull base centers [109].

#### 5.2.2. Primary Radiotherapy in Large VS

As stated earlier, surgery is often the preferred treatment option in large VSs. Reported data on radiotherapy without any prior intervention for large VS is limited. Among those studies, Langenberg et al. reported the result of 33 patients with large unilateral VS exceeding the volume of 6 cm^3^ and primarily treated by GK (mean prescribed dose = 12.6 Gy) [110]. After a median FU of 30 months, 88% of the cases had achieved radiological growth control. One year after the treatment all patients had a good FN function. Seven out of 12 patients who had serviceable hearing before treatment kept their functional hearing. After GK, ventriculoperitoneal shunt was placed in two patients because of hydrocephalus and two patients experienced Grade 2 transient facial paresis. No major complications were seen nor treatment-related mortality. Bailo et al. reported on GK as primary treatment (median marginal dose = 13 Gy) in 59 patients having large VS (mean volume = 5.98 cm^3^) [111]. They achieved 100%, 97.9% and 97.9% tumor control rates at 3, 5 and 10-years, respectively. Overall, hearing preservation after a mean follow-up of 70.4 months was 31.3% in 16 patients. Treatment-related complications included three patients with new permanent FN deficit, four patients with new or worsened trigeminal nerve impairment and hydrocephalus requiring a shunt was observed in 10 patients. A very recent study by Lefrance et al. included a larger series of 86 VS patients followed up for a mean period of 6.2 years (range; 3–16 years) [112]. All patients underwent GK and the mean prescribed dose was 11 Gy. Recurrence was seen in eight patients (tumor control rate = 90.7%). Twenty-five out of 38 patients kept their hearing after GK. Interestingly, no radiation-induced toxicity was observed. Despite the risk of acute transient swelling that may result after irradiating large VSs and potentially affect the brainstem and CNs, results from abovementioned studies show that radiotherapy can be safe and effective as a first-line treatment in cases where surgery is not the preferred treatment.

### 5.3. Planned Radiotherapy After Subtotal Resection in Large VS

**Figure 3:**
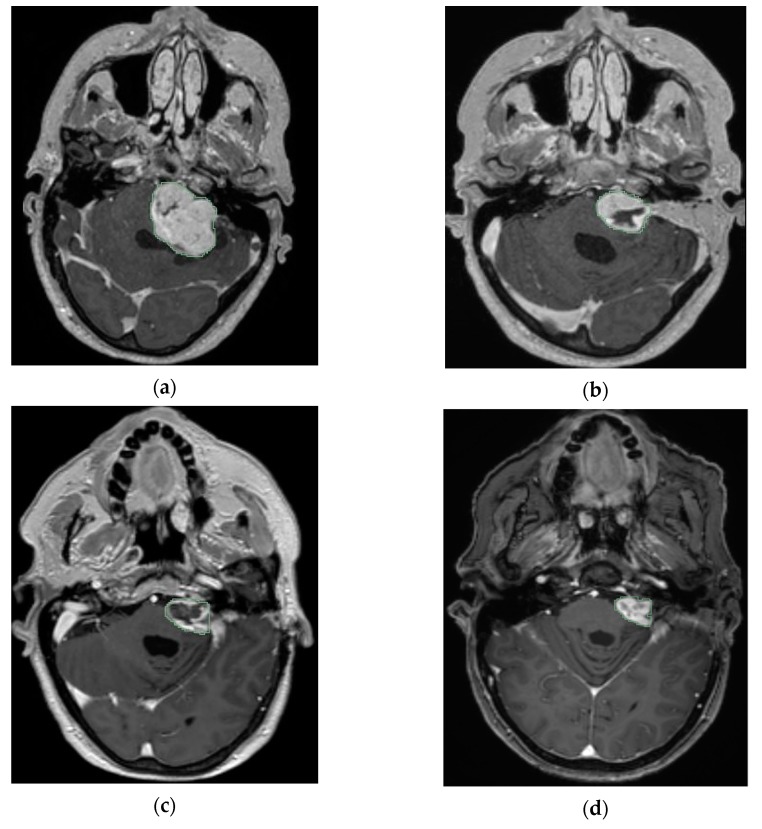
An axial T1 MRIs with contrast for left VS (KOOS D), showing tumor shrinkage after the combined approach; A, is the image before surgery; B, after surgery and before GK; C, 6 months after GK; D, 5 years after GK (courtesy of Y. Temel).

Over the past decades, surgery has been considered as the preferred treatment in patients harboring large VS [113,114]. In recent years, there is a trend towards the approach of planned SRS after planned subtotal resection (Figure 3).

The theory behind planned combined therapy is a safe resection minimizing the risk of FN injury and better control of the residual tumor by applying a dose of radiation [115,116,117]. Data related to the radiation type, dose delivered, recurrence, radiation-induced side effects and functional outcomes are summarized in Table 2.

In 2003, Lwai and his colleagues published the first series using the approach of planned partial resection followed by GK [118]. They analyzed 14 patients harbored tumors with 30 mm and larger (two patients were suffering from NF2). The mean time between surgery and GK was 2.9 months. The tumor residues (mean = 18.9 mm) were irradiated with doses ranging from 10 to 14.1 Gy (mean = 12.1 Gy) and followed up for 12–72 months (mean = 32 months), respectively. After GK, the tumor size decreased in six patients, did not change in five patients and increased in three patients (meaning 79% growth-control). No radiation-related injuries were seen. In the final follow up, an excellent FN function was achieved in 85.7% of the patients and the tumor growth control rate was 79%.

In 2006, Park et al. reported a 100% growth control rate for eight patients after removing more than 90% of the tumor followed by GK [119]. A dose of 12 Gy was delivered to the remnant tumor (average size = 4.6 cm^3^) and the mean follow up period was 68.8 months. FN preservation was found to be inversely proportional to the extent of tumor resection. No deterioration was seen in the FN functions after GK. 

In 2007, Yang et al. described 61 patients treated by GK (mean dose = 12.5 Gy) after planned subtotal resection (mean residuals’ volume = 3.68 mL) [120]. They achieved 96.5% and 93.5% tumor control rates after 4 and 8-years, respectively. Within four years after GK, tumor progression was seen in three patients. However, no toxicity or acute radiation-induced effects were seen due to GK. At the last follow up, FN function was preserved in 95% of the patients and only 30% maintained their hearing. In this study, the numbers of 24 patients harbored a cystic VS and 26 a solid VS Analysis showed that cystic VSs required less time to shrink than solid VS, with mean half reduction times equal to 2.58 and 14.34 years, respectively.

In 2008, Fuentes et al. reported similar results to those of Park et al. (2006). They included eight patients with a VS diameter ranging from 35 to 45 mm [121]. Nine months after surgery remnants (mean volume = 1.16 cm^3^) were irradiated with doses ranging between 22 and 26 Gy. The analysis showed that none of the patients developed a relapse (100% control rate) or radiation-induced complication. At the last follow-up, the FN function was preserved in 87.5% of patients.

In 2011, Van De Langenberg and coworkers evaluated 50 patients who had undergone the combined approach [115]. The median follow up time was 33.8 months. The FN function was preserved in 94% of the patients. No high grades of toxicity were documented after GK. The observed radiation-induced effects were a grade II transient facial paresis in two patients, transient trigeminal hypesthesia in one patient and one patient presented with persisting FN spasms. 

In 2012, Pan et al. also shared their experience with the combined treatment strategy in 18 patients [122]. A 100% tumor control rate at a mean follow up of 57.7 months was found. Eleven out of eighteen patients presented with serviceable hearing before surgery and none showed deterioration after treatment with no difference between surgery and GK. Moreover, the FN function was preserved in 89% of patients. No adverse effects were seen after GK except one case of transient hearing loss.

In 2015, Lawi et al. published a different cohort than the one in 2003 treated by the same strategy [117]. Forty VS patients were included in the analysis with a diameter of 25 mm and above. Following surgery, residual tumor was irradiated with a median dose of 12 Gy. Four patients developed recurrence after 12–40 months. Tumor growth control rates were 92%, 86% and 86% at 3, 5 and 10-years, respectively. At the last follow-up, 95% of the patients had a good FN function. With respect to hearing, 42.9% of the patients who had serviceable hearing before surgery preserved hearing. No brain injuries or permanent complications such as facial palsy or trigeminal neuropathy were seen after GK.

In 2017, Daniel et al. shared their experience with the combined approach [123]. Thirty-two patients harboring large VS (Koos IV) were included in the cohort analysis. Residual tumors were irradiated with a mean dose of 12 Gy. Three patients showed tumor recurrence within 3 years (91.1 % control rate). No neurological deficits were seen after GK treatment. Preservation of facial and cochlear nerve function was 100% and 76.9% (10/13), respectively.

### 5.4. Radiation-Induced Effects After Radiotherapy in Both VS and NF2 VS

Irradiating VS and NF2 VS may induce several early or acute effects. One is gait disturbance because of vestibular dysfunction or hydrocephalus [124]. Brain necrosis can result from irradiation, especially in the case of large tumor volumes or repeated irradiation [125]. In a recent study with 235 VS cases undergoing GK, the following complications were documented after GK: pseudo-progression in 43 patients, facial myokymia in 25 patients, trigeminal neuropathy in 22 patients, hydrocephalus in 15 patients and vertigo in 14 patients. A marginal dose ≥ 13 Gy was associated with a high probability of losing serviceable hearing and a high probability of vestibular nerve dysfunction [126]. Due to the histopathological nature of NF2 tumors and since NF2 patients are younger, they are more likely to have a higher risk of developing radiation-induced effects than sporadic VS patients [127,128,129].

A main long-term radiation side effect can be adhesion formation, which makes salvage surgery challenging in case of treatment failure [130]. Secondly, malignant transformation or the induction of secondary neoplasm can theoretically occur. Reporting on radiation-induced tumors must consider Cahan’s criteria, which take into account the following aspects. First, a latency period must be endorsed between the irradiation and the formation of the second tumor. Second, the presence of the new lesion must be seen within the field of the original irradiation. Third, the new tumor must be different histologically from the original tumor. Fourth, the absence of any genetic syndrome that makes cancer susceptible to the formation [131]. In 2014, Patel and Chiang reviewed 36 cases of SRS-induced neoplasms. Of these 36 cases, 22 were initially diagnosed with VS. Overall, they estimated the chance of developing secondary neoplasm post SRS at 15 years to be 0.04% [132]. In a recent review (that included 9460 unilateral VS in the analysis) 66 patients experienced a secondary tumor after treatment, of which only six cases were malignant. The estimated time to the second neoplasm formation post VS treatment was found to be 0.8% at 5-years [133]. In regards to patients with NF2, Balasubramaniam et al. have reviewed 20 reported cases of secondary neoplasm or malignant progression of benign primary tumors linked with SRS or FSRT. Most of these cases (14 out of 20) were VSs with eight being NF2 patients without other significant risk factors [134]. Another study has included a bigger NF2 cohort (118 patients), aiming to assess retrospectively the risk of radiosurgery on tumors associated with suppressor genes. Only one NF2 case was identified with glioblastoma 3 years after radiosurgery [135]. Although a definite carcinogenic risk factor associated with NF2 population is yet to be known, they should be considered with extra care in treatment planning.

## 6. Medical Therapy and NF2 VS

As stated earlier, tumor control and functional outcomes are the chief considerations when managing both VS and NF2 VS; but the challenge is harder with NF2. To overcome this dilemma, intravenous chemotherapy with Bevacizumab (BZA) has been introduced in the last decade as one of the treatment options for NF2 patients. BZA works as a monoclonal antibody against the vascular endothelial growth factor [136]. The use of BZA was initially proposed by Plotkin et al. and offered to 10 NF2 confirmed patients whom they have an evidence of tumor progression and considered poor candidates for surgery and radiotherapy or refused these treatments. The efficacy of BZA was shown as tumor shrinkage in nine patients and hearing improvement in four out of seven patients presented with hearing response prior to treatment. No serious adverse effects were seen and none of the patients discontinued the treatment due to the reported side effects (grade 1 and 2) [137]. Following that, a number of studies reported in the influence of BZA to decrease tumor volume and improve the hearing response in NF2 patients [138,139,140]. Although, the existing evidence supporting the use of BZA as a medical therapy is not stronger than level 3 at this moment [141]. To broaden the view, a recent systematic review aimed to evaluate the safety and the treatment outcomes of BZA with NF2 patients has included 161 patients from eight articles in their pooled analysis. Results showed tumor shrinkage in 41%, stability in 47% and progression in 7%. The hearing response in patients with assessable audiometric data was improved in 20%, no change in 69% and deterioration in 6%. Cardiac hypertension and renal proteinuria are the most frequently reported side effects (33% and 43%, respectively), and 17% of these percentages were in serious grades (Grade 3 and above) [142].

Another promising way of managing VSs is by inhibiting cell surface receptors and intracellular signaling pathways [143]. As mentioned earlier, mutations of NF2 gene that encode the tumor suppressor protein “merlin” are inactivated in most of sporadic VSs and NF2 VSs. Following the loss of merlin, an array of mitogenic signaling cascades that are responsible for tumorigenesis, proliferation and survival will be abnormally activated. Then, a deregulation of key signaling pathways such as mTORC1, Ras/Rac and EGFR is known to occur [24,144,145]. Therefore, some of the signaling pathways inhibitor drugs such as that used to treat renal cell carcinoma patients (Everolimus) were tested with NF2 patients [146]. Although everolimus did not induce shrinkage of VS, the tumor seemed to stabilize and the treatment was well tolerated. Others recently suggested the efficiency of combination of mTORC1/2 inhibitor AZD2014 and the tyrosine kinase inhibitor dasatinib in reducing metabolic activity of NF2 VS [147]. Although the U.S. Food and Drug Administration is yet to approve any drug for VS or NF2, continuous developments towards this approach are worthwhile trials.

## 7. Conclusions

In summary, loss of NF2 merlin function due to mutation was associated with sporadic and NF2 VS. Patients with NF2 VS generally inherited the mutation from the affected allele of a parent. Although more than 20 genes have been reported to be associated with sporadic VS, in familial cases (except for NF2 gene) other genetic mutations have rarely been reported.

The management of large VS in both VS types include surgery with or without adjuvant radiation. When making a treatment decision it is important to take into account individual needs, tumor characteristics as well as the given possibilities of the care provider facility. The management for the medium- or small-sized VS includes wait and scan, radiotherapy and/or surgery. In recent years, there is a trend towards the approach of planned SRS after planned subtotal resection. The induction of secondary neoplasm as a late side effect after radiotherapy seems to be a negligible risk and drug-based approaches are arising as new therapeutic options.

## Figures and Tables

**Table 1:** The comparison of sporadic VS and NF2 VS.

Characteristics	Sporadic VS	NF2 VS
Mutation in the NF2 gene	60%	90%
Common type of mutation	Point mutations and small deletions	Truncating mutations
The “hit”hypothesis	One hit, two hits	One hit, two hits, four hits
Family history	No	Approximately 50% of individuals with NF2 have an affected parent
Tumor location	Unilateral	Bilateral
New cases	95%	5%
Concomitant tumors	No	Meningiomas, astrocytomas and ependymomas
Standard diagnosis technology	MRI	MRI
Age of onset	Middle-age	Childhood
The preferred management	medium- or small-sized: wait and scan, radiotherapy and/or surgery large size: surgery	medium- or small-sized: wait and scan, radiotherapy and/or surgery large size: surgery
Ophthalmological lesions	No	Cataracts, epiretinal membranes, retinal hamartomas
Cutaneous lesions	No	Skin tumors, skin plaques, subcutaneous tumors

**Table 2:** Extracted data from studies found reporting on planned radiotherapy after subtotal resection in large VS.

Author	Patient Number	Tumor Diameter OR Volume Before Surgery	Tumor Diameter OR Volume After Surgery	RS Type	Prescribed Dose	Interval between S and RS (Months)	Follow Up (Months)	Control Rate	Regrowth	Time to Manifestation (Months)	RS Side Effect	FN Function (Preservation)	Hearing Function (Preservation)
Iawi Y Yamanaka, and Ishiguro (2003)	14	Diameter ≥ 30 mm	Mean diameters = 18.9 mm (9.8–36.1 mm)	GK	Mean = 12.1 Gy (10 - 14.1 Gy)	1–6 months (mean = 2.9 months)	Mean = 32 months (12–72 months)	79%	3 (2 with NF2 and 1 with extra-large VS	^1^NM	No complications	85.70%	Among 3 patients with useful hearing (with G&R Class 1 and 2 preoperatively), postoperatively useful hearing was preserved in only 1 patient.
Park et al.(2006)	8	Volume ≥ 3 cm^3^	Mean volume = 4.6 cm^3^	GK	Mean = 12 Gy	1 Week- 6 months	68.8	100%	0	NM	No complications	31 patients underwent R-STR and 8 of them received GK. The overall FN preservation rate = 87%	NM
Yang et al. (2007)	61	Mean volume = 20.6 ± 11.1 mL (range: 18–67)	Mean volume = 3.68 mL (range: 0.52–15.50 mL)	GK	Marginal doses 9–14 Gy (mean: 12.5 GY)	Median = 5.8 months	After KG, median follow up = 53.7 months (range 24.1–102.2)	The 4- and 8-year actuarial tumor control rates were 96.5% ± 2.4% and 93.5% ± 3.7%, respectively	3 patients	Within 4 years after GK	No complications	95%	Before surgery, only 10 out of 61 patients had serviceable hearing (grade 1 and 2). After surgery, 5 out of 10 had serviceable hearing. At the last follow up and after GK, 3 of the 5 patients had serviceable hearing (30% : 3/10)
Fuentes et al. (2008)	8	Mean diameter = 40 mm (35–45)	Mean volume = 1.16 cm^3^ (0.3 - 2.2 cm^3^)	GK	The mean peripheral dose = 11.8 Gy (range: 11–13 Gy). Mean dose to the tumor center = 23.75 GY (22–26 Gy).	Mean = 9 months (6–12 months)	Mean follow-up time after the GK = 46 (range: 12–73) months.	100%	0	NM	No complications	87.5%	NM
Van De Langenberg et al. (2011)	50	Mean volume = 14.9 cm^3^ (4.1–36.1)	Mean volume = 3.34 cm^3^ (0.22–11.8 cm^3^)	GK	Mean dose prescribed to the isodose covering 90% of the tumor = 12.9 Gy (12 - 13). Mean maximum dose = 21.1 Gy (18–26). The mean tumor margin dose = 11 Gy (9.4 - 11.9).	Mean = 8.5 (2 - 24)	Median = 33.8 (12 - 84)	Clinical tumor control = 92%, radiological tumor control = 90%	4	Mean = 31.5 months ( 22–49)	HB Grade II transient facial paresis developed in 2 patients. One patient developed transient trigeminal hypesthesia. One patient experienced persisting FN spasms.	94%	Before surgery: All patients reported hearing loss, and only 4 (8%) of the 50 patients presented with serviceable hearing, all Class B.
Pan et al. (2012)	18	Mean volume = 17.5 cm^3^	Mean volume = 9.35 cm^3^	GK	12 Gy	Mean = 3.6	Mean = 57.7 (at least 3 years)	100%	0	NM	1 patient had transient hearing loss following GK.	89%	100%/Before surgery, 11 patients had serviceable hearing and all were preserved.
Iwai et al. (2015)	40	Median diameter = 18.6 mm (9.1–27.1)	Median volume = 3.3 cm^3^ (0.4–10.4)	GK	Median dose = 12 Gy (10–12 Gy)	Median = 3 (1–12)	Median = 65 (18–156)	At 3 years = 92%, at 5 years = 86%, at 10 years = 86%	4	After 12, 27, 34 and 40 months	• 2 patients suffered transient facial spasms. • 2 patients experienced transient trigeminal neuropathy	95%	Before surgery: 29 patients had some hearing preservation. At the last follow-up: 42.9% (6/14) preserved their hearing
Daniel et al. (2017)	32	Mean volume = 12.5 cm^3^	Mean volume = 3.5 cm^3^	GK	Mean dose = 12 Gy	Mean = 6.3	Mean = 29	91.6%	3	After 2.6, 2 and 1.2 years	No complications	100%	Before surgery: 13 had normal hearing, After treatment 10 preserved hearing functions 76.9%

NM: not mentioned.
